# The Prescription Opioids and Depression Pathways Cohort Study

**DOI:** 10.20900/jpbs.20200009

**Published:** 2020-04-28

**Authors:** Jeffrey F. Scherrer, Brian Ahmedani, Kirsti Autio, Lynn Debar, Patrick J. Lustman, Lisa R. Miller-Matero, Joanne Salas, Scott Secrest, Mark D. Sullivan, Lauren Wilson, Sarah Skiold-Hanlin

**Affiliations:** 1 Department of Family and Community Medicine, Saint Louis University School of Medicine, 1402 South Grand Blvd, St. Louis, MO 63104, USA; 2 Center for Health Policy and Health Services Research and Behavioral Health Services, Henry Ford Health System, One Ford Place, Detroit, MI 48202, USA; 3 Kaiser Permanente Washington Health Research Institute, 1730 Minor Ave, Seattle, WA 98101, USA; 4 Department of Psychiatry, Washington University School of Medicine, 660 South Euclid Ave. St. Louis, MO 63110, USA; 5 Department of Psychiatry and Behavioral Science, University of Washington School of Medicine, Seattle, WA 98195, USA

**Keywords:** epidemiology, pain, opioids, depression, anxiety, substance use disorder, sleep, health related quality of life, social support

## Abstract

**Background:**

Results from studies using medical record data indicate chronic (>90 days) opioid analgesic use (OAU) is associated with new depressive episodes (NDE), worsening depression and risk for depression recurrence. This body of evidence is based on retrospective cohort studies and medical record data. Limitations of existing research are overcome in a new prospective cohort study of the opioid-depression relationship.

**Methods:**

Prospective cohort of 1500 adult patients recruited from two health care systems. Eligible subjects started a new period of OAU and have 30 to 90 days of OAU at baseline. Diagnostic assessments for psychiatric disorders, structured measures of pain, pain functioning, opioid use, social support, sleep and impulsivity will be obtained at baseline, 6-month and 12-month follow-up. Baseline participants will be invited to 12 monthly brief assessments of pain-related functioning, depression symptoms and opioid use.

**Innovation:**

Robust control for confounding by indication and detailed phenotyping of depression and opioid use disorder.

**Anticipated results:**

Chronic OAU will be associated with new onset of a depression phenotype characterized by anhedonia and somatic symptoms. This relationship will be partly, but not completely explained by impaired functioning and low social support.

**Conclusions:**

Although the annual number of opioid prescriptions in the United States has decreased, over 190 million patients have OAU each year. If chronic OAU leads to a clinically meaningful affective disorder, independent of pain, then we need to consider depression an important adverse effect of chronic OAU and adjust care for chronic pain accordingly.

## INTRODUCTION

There is substantial evidence that depression has a fundamental role in the prescription opioid epidemic [1]. The bi-directional association between chronic pain and depression is well-documented. Patients with chronic pain often see pain as the cause of their depression and seek opioid therapy to reduce both. The bi-directional association between chronic prescription opioid analgesic use (OAU) and depression has been suggested by previous observational studies [2–8] and is the foundation for our prospective cohort study. We report on a protocol that is designed to confirm this bi-directional link and determine if a bi-directional pathway is independent of pain severity and pain related functioning, social support, substance use disorders, comorbid anxiety disorders, impaired sleep, sleep apnea and impulsivity.

Depression, and common comorbid conditions such as anxiety and posttraumatic stress disorder, are related to increased likelihood of patients with pain receiving opioids, receipt of higher opioid doses, longer duration of opioid use and increased risk for opioid misuse [7,9–12]. A review of psychopathology in pain [1] suggests that the OAU epidemic in the United States reflects under-detected and under-treated mental illness in patients with non-cancer chronic pain. Patients with depression report greater pain severity and are less likely to experience pain relief from OAU compared to non-depressed patients [13,14]. The blunted analgesic response to OAU may explain why depressed patients with chronic pain are more likely to receive higher morphine equivalent doses (MED), which in turn increases risk for opioid misuse and abuse, accidental overdose and death. Depression may be related to greater OAU in non-cancer pain because patients overuse opioids in an attempt to reduce anxiety, moderate mood and improve sleep [9].

A number of retrospective cohort studies have found longer, (≥90 days) opioid analgesic use (OAU) and rapid dose increases are associated with significantly greater risk of developing new depression episodes (NDE) [3,5,15–17]. Patients starting opioids vs those without opioid use have an increased risk for depression recurrence [18] and chronic OAU is associated with worsening existing depression [4]. These results were independent of non-cancer pain. There are few existing prospective cohort studies of new prescription OAU that measured depression as a key outcome. Results from the Pain and Opioids IN Treatment (POINT) study, a prospective cohort study of new prescription opioid users, revealed that among the 61% of subjects with depression in their lifetime, 48% had depression onset after opioid initiation [19]. Those who did develop depression, compared to those who did not, started opioids at a younger age, had less social support and more opioid related difficulties [19]. A study of middle-aged and older patients found no association between longer OAU and new onset depression, but opioid related negative mood was reported by 21% of those who dropped out of the study [20]. At 12-month follow-up, those with more regular, higher dose use had more depression symptoms than those with minimal or no OAU [20]. Last a 2-year prospective cohort study of stable long-term opioid users found no risk for depression following dose escalation, however this study differs from others in that the sample was not starting a new period of opioid use at baseline and were all stable, long-term users [21].

With few prospective cohort studies, the existing evidence for a bi-directional association between OAU and depression is based on retrospective medical record data or longitudinal studies without detailed measures of depression and comorbid psychiatric disorders. It is possible that OAU worsens depression symptoms or elicits recurrence of psychiatric disorders present before the start of observed medical record data. Studies of chronic OAU and NDE in medical record data lack granularity necessary to conclude that OAU leads to NDE or is better described as leading to a mix of depression, dysthymia, anhedonia and vital exhaustion.

**Figure 1.**
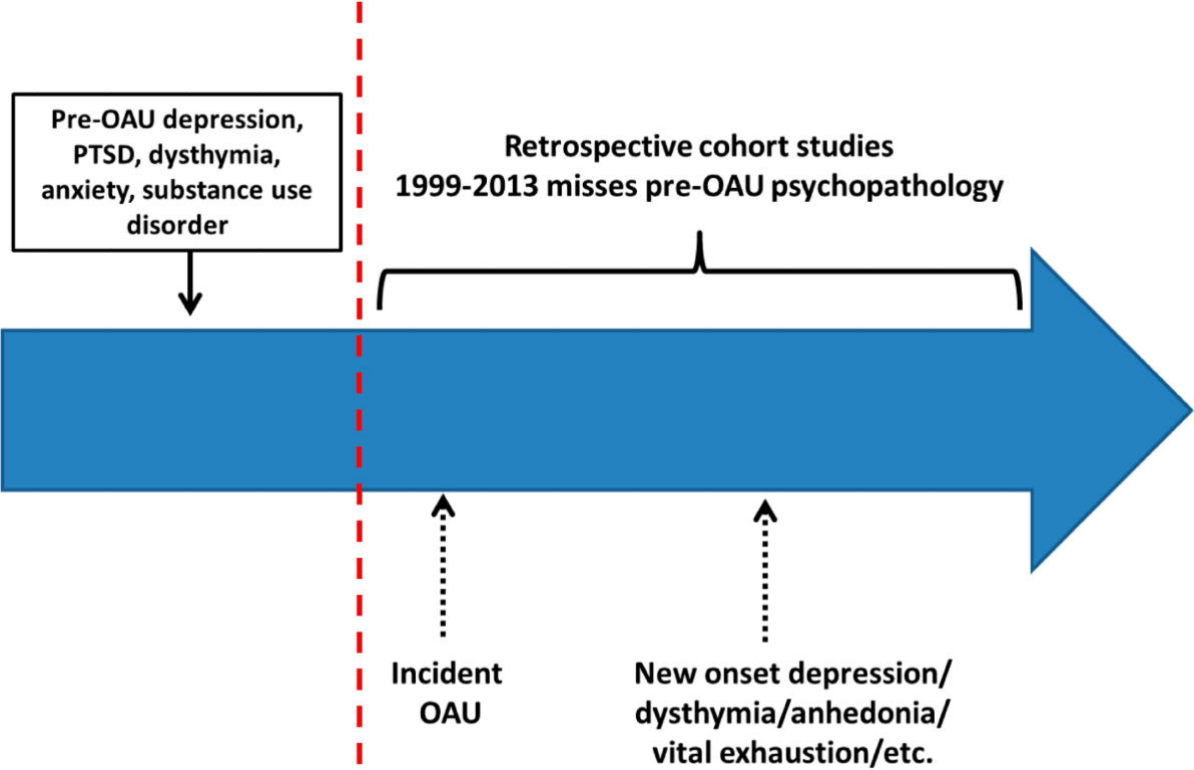
Factors preceding OAU may increase risk for new onset depression.

Therefore, the first aim of the Pathways study is to determine if factors that precede a new period of OAU, as shown in Figure 1, explain the association between chronic OAU and new onset depression and other affective disorder phenotypes. If NDE is explained by OAU alone and not by pre-existing risk factors, then the opioid epidemic is generating new cases of depression in a large population of middle-aged adults. We test whether the OAU-NDE association is moderated by pre-existing depression, posttraumatic stress disorder and substance use disorder (SUD), including opioid use disorder.

The overall goal of the Pathways study is to reveal why some, but not all patients with chronic OAU, develop NDE. Thus, our second objective will determine if common consequences and correlates of OAU such as poor pain-related functioning, impaired sleep, and/or substance use disorder are related to increased risk of NDE, independent of OAU. This aim will help generate possible explanations as to what pathways may exist from OAU to NDE.

A major innovation of our study is measuring dysthymia, mania, anhedonia, vital exhaustion and major depressive episodes to determine the phenotype most likely to occur following chronic OAU. Subsequently, we will be able to identify which depression phenotypes and patterns of comorbidity are most strongly associated with subsequent opioid misuse, problem use and use disorder. To our knowledge, there are no studies which have captured this range of affective disorders/syndromes and this breadth of opioid misuse and abuse/dependence.

The last objective in the Pathways study is to understand if the patterns of co-developing pain and OAU are associated with course of depression (stable, worsening, improving). If depression decreases in patients with increasing, decreasing or stable pain that co-develops with decreasing OAU, then decreasing OAU exposure, not pain, should be a means to reduce the burden of persistent depression in non-cancer pain populations. Monthly data collection will be used to compute trajectories of depression that can be linked to changes in OAU, function and pain. This objective expands seminal work by Kroenke et al. [22], which revealed a reciprocal, longitudinal relationship between depression and chronic non-cancer pain but did not account for the co-development of chronic OAU.

## MATERIALS AND METHODS

### Subjects

Baseline recruitment continues until 1500 subjects are enrolled. Feasibility of recruiting this sample and conducting repeated follow-up assessments is enhanced by splitting the target sample between Saint Louis University’s (SLU) academic medical practice and Henry Ford Health System (HFHS). Eligible subjects (eligibility described below) will be identified using health care systems’ electronic health records (EHR). By using targeted recruitment and with IRB approval of a HIPAA waiver, we will be able to measure potential non-response bias by comparing key EHR data among participants and refusals.

Subjects are eligible if they are 18–70 years of age, free of cancer and HIV and have started a new period (i.e., no opioid prescriptions in the past 3 months) of OAU. We recruit patients who have 30 to 90 days of OAU at baseline. Those with less than 30 days of OAU are not eligible because there is no evidence for an increased risk for NDE. Our electronic health record algorithm generates an eligible sample enriched for chronic (>90 days) opioid users. Prescription OAU includes short and long-acting formulations and any dose of codeine, tramadol, hydrocodone, oxycodone, morphine, methadone, fentanyl patch, dihydrocodeine, hydromorphone, levorphanol, meperidine, oxymorphone, pentazocine and tapentadol.

At baseline, OAU eligibility criteria are confirmed and subjects consent and release PHI prior to beginning the survey. All assessments are administered in RedCap (a programmable internet based survey system) and either completed by the subject on the internet or by a telephone interviewer entering answers into RedCap. Data is collected at baseline, 6-month and 12-month follow-up. Those who complete baseline are invited to complete 12 monthly, brief surveys which can be completed by the subject using RedCap or by telephone interview.

All instruments used for study constructs, when they are administered, and their sources are shown in Table 1. Baseline and 12-month assessments measure the following constructs: (1) chronic pain, pain severity, pain related impaired function, pain duration and recency; opioid medication use and non-opioid pain treatments; (2) DSM criteria lifetime and past year major depressive episode, dysthymia, mania, opioid use disorder, heroin use disorder, cocaine/stimulant use disorder and marijuana use disorder. To limit subject burden, we use shorter instruments to measure lifetime and current PTSD, generalized anxiety disorder and current anhedonia and vital exhaustion, alcohol misuse, smoking and subject report on parental history of depression and substance use disorder; (3) sleep quality and sleep apnea; (4) social support and social functioning and (5) sociodemographics. The EHR data will be used to measure comorbid conditions, use of other medications and any pharmacological and non-pharmacological treatments provided for pain and other conditions. The 6-month follow-up is reduced by excluding DSM assessments. Baseline and 12-month follow-up take between 45–60 min to complete and the 6-month follow-up will take 30–40 min to complete. Each monthly assessment includes the PHQ-9, self-reported OAU and pain related impairment.

We programmed the Semi-structured Assessment for the Genetics of Alcoholism (SSAGA) [23] diagnostic instrument to obtain DSM-IV assessments. To our knowledge, this represents the first, web-based, self-administered diagnostic instrument applied in clinical epidemiology. The SSAGA has good test-retest reliability (kappa range 0.70–0.90). Comparison of SSAGA derived diagnosis to diagnoses obtained from the Schedule for Clinical Assessment in Neuropsychiatry (SCAN) revealed sensitivity and specificity of 88.2 and 82.5, respectively for depression, 80% sensitivity and 95.7% specificity for opioid abuse/dependence, and 73.3% sensitivity and 86.1% specificity for cannabis abuse/dependence [24].

Participants are provided a $50 incentive for baseline, 6- and 12-month follow-up survey completion and $10 for each of the 12 brief monthly surveys completed. A $1000 raffle is offered to those who complete baseline, 6-month follow-up and the first 6 brief monthly surveys and a second $1000 raffle offered after the 12-month follow-up for those who complete all assessments. The electronic health record algorithm identifies eligible subjects on a weekly basis. Recruitment packets are sent to eligible subjects who are encouraged to use the internet to complete the survey. Subjects who do not participate after receiving the recruitment packet are contacted by research staff via telephone calls. Attempts to recruit continue for 5 weeks after sending invitations to participate. Beyond 5 weeks, subjects are no longer eligible because the early phase of the new period of OAU is past. Standard thank-you and reminder cards are used to enhance retention.

All data is stored with a code number and after data collection is complete, coded data is sent to SLU for data analysis. The procedures described have been approved by the SLU and HFHS Institutional Review Boards.

### Analytic Approach

Prior to analysis, we will first evaluate missingness and non-response bias. Missingness will be handled with multiple imputation techniques. We will test for non-participation bias as a function of age, race, gender, psychiatric and pain diagnoses and analgesic prescription obtained from the EHR. Analyses will be weighted by stabilized inverse probability of participation weights [42–49], if weighted and unweighted results do not differ, then we report results using unweighted data.

If differences are observed by data collection modality, we will use a dummy variable in all analyses that indicates self-administered vs. telephone administration. To control for clustering and reduced standard error related to subjects within the same health care system, we will apply a Taylor Series linearization variance estimation, which is similar to generating robust standard errors using sandwich variance estimation [50].

Because opioids are prescribed for pain and pain is associated with depression and other outcomes, we control for confounding by indication using propensity scores (PS) and inverse probability of treatment weighting (IPTW) [42–45]. In this instance, we balance confounding factors across OAU durataion, i.e., OAU ≤ 90 days and OAU > 90 days. We have used this method successfully in prior studies to balance confounders by OAU duration when studying duration of OAU and risk of NDE and worsening depression [4,15,18].

To determine if pre-OAU factors account for post-OAU NDE, we will use the subject reported NDE age onset. The main exposure is defined as ≤90 days vs >90 days OAU that occurs prior to NDE. Pre-OAU effect modifiers will be measured at baseline. Unweighted and IPTW, modified Poisson regression models using robust error variances will calculate relative risk of NDE comparing OAU > 90 vs ≤ 90 days [51]. Separate, stratified models based on pre-OAU lifetime depression, PTSD, anxiety, etc. will be calculated to assess relative risk of NDE based on OAU in the absence/presence of pre-OAU comorbid psychiatric disorders. A non-stratified model will include interaction terms for OAU x pre-OAU comorbidities to determine if these pre-OAU factors significantly modify risk of NDE.

To determine whether post-OAU factors are related to increased risk of NDE independent of OAU, we will select subjects without any indication of depression from baseline through 12-month follow-up. Measurements of post-OAU factors like opioid misuse, social support, pain function, and poor sleep, will be computed from 6-month follow-up data. OAU duration of ever >90 vs ≤90 days will be computed from baseline and 6-month follow-up data and will occur prior to post-OAU factors. Modified Poisson regression models with robust error variances will be used to calculate relative risk of NDE. Separate models for each post-OAU variable will be computed. First a crude model and then an adjusted model adding only OAU duration are computed to assess whether post-OAU variables have an effect independent of OAU duration. A fully adjusted model will include all post-OAU variables and OAU duration.

Brief monthly assessments are used to measure patterns of pain and opioid co-development associated with stable, increasing, decreasing and no depression symptoms. Monthly measures will be collapsed into 4 quarterly time points by averaging measures such as opioid dose. This allows for patients to miss a monthly measure while still providing informative data for 4 time points. Following Wiggins et al. 2015 [52], we use complementary approaches by first using a person-centered approach with parallel process latent class growth analysis (PPLCGA) [53,54] to measure co-development of pain severity/function, OAU dose, and depression over time. PPLCGA provides information on the extent to which pain, OAU, and depression change concurrently over time by classifying individuals to qualitatively different homogenous groups based on initial levels (intercepts) and changes over time (slopes). Models will be estimated starting with one class and increasing classes until model fit and interpretability are maximized. Fit indices include the Lo-Mendell-Rubin likelihood ratio test, Vuong-Lo-Mendell-Rubin, AIC, BIC, sample size adjusted BIC, entropy and minimum class size of 1%. The second approach is variable-centered and helps understand the dynamic interplay of OAU, pain, and depression, in terms of reciprocal contributions over time. An autoregressive cross-lagged model with OAU dose, pain, and depression at quarters 1, 2, 3 and 4 will regress each variable on all other variables at the previous time point [55,56]. These models assess: (a) an auto-regressive component, or continuity of each construct; and (b) a cross-lag component, or the relationship of each variable at each time point with the other variables at the prior time point. Model fit will be assessed with likelihood ratio chi-square tests, comparative fit index, root mean square error of approximation and standardized root mean square residual.

Depression phenotypes and opioid misuse phenotypes are computed using Latent Class Analysis (LCA) [57]. Separate LCA will be computed using 12-month follow-up data to classify individuals based on: (1) NDE symptoms and (2) NDE comorbid with SUD (alcohol, drug, opioid). The number of classes will be determined based on fit indices, including sample size adjusted BIC, adjusted AIC, and Lo-Mendell-Rubin likelihood ratio test as well as interpretability of classes [58]. The relationship of OAU duration and each latent class profile at 12-month follow-up will be assessed using separate unweighted and IPTW multinomial logistic regression (>2 classes identified) or binary logistic regression (2) classes identified), with the latent class profile as the outcome.

To determine which depression subtypes and comorbidity subtypes in patients with >90 day OAU are most strongly associated with incident opioid misuse and opioid use disorder, we will select subjects without pre-OAU opioid misuse who have >90 days OAU ever in the 12 months after baseline. Outcomes include: (1) incident opioid misuse and abuse, defined as the first occurring at either 6-month or 12-month follow-up; and (2) opioid use disorder defined at 12-month follow-up. Separate baseline exposures are: (1) depression sub-types; (2) depression with comorbid SUD; and (3) depression phenotypes such as DSM-4 major depressive episode vs dysthymia vs bipolar and anhedonia vs. vital exhaustion. Separate LCA’s on baseline data using MPlus 8.0 will define latent class profiles for depression sub-types and depression comorbidity profiles. Covariates occurring in all assessment time points relative to the outcome of interest will be included in models. Binary logistic regression analyses will estimate the association of each exposure with each outcome by calculating separate crude and adjusted models in SAS v9.4.

### Limitations

Our design is intended to enroll patients who are starting a new period of prescription OAU and many patients will have had a prior OAU episode. Some subjects may be opioid naïve, but the total sample will not be directly comparable to a cohort of opioid naïve patients. We chose this approach, in tandem with recruiting subjects with over 30 days of OAU, to increase the proportion of subjects who will become long-term opioid users. If we recruited opioid naïve patients, most patients would be short term users with little risk for adverse outcomes. Our assessment of depression obtains lifetime and current depression using a diagnostic interview with dates of onset and recency. We modified the SSAGA diagnostic interview to allow for self-administration. Thus stem items are worded for self-administration and it is possible that psychometric properties could differ from the standard SSAGA.

Social desirability could influence accuracy of self-reported prescription OAU, however Goesling et al. 2015 [59] obtained an 83% sensitivity and 94.2% specificity when comparing subject self-reported prescription OAU to chart abstraction. Recall-bias is a potential limitation when measuring lifetime events and symptoms. Our recruitment method, obtaining measures closest to the start of a new period of opioid use is intended to limit recall bias, but we acknowledge that there is always a risk of recall-bias in prospective cohort studies.

## DISCUSSION

The Pathways study offers an opportunity to observe the relationships between prescription OAU, pain, depression and comorbidities and psychosocial factors in a prospective cohort study in which subjects are starting a new OAU period. An important distinction of our study is that we are creating a cohort of prescription opioid users and not illicit opioids or heroin users. Our design controls for a history of illicit substance use disorder when measuring the association between OAU and depression. To our knowledge there are no similar cohorts in the United States. The Australian Pain and Opioids IN Treatment (POINT) study [60,61], praised for its novel longitudinal design [62], shares some elements of our cohort study and is meant to reveal predictors of OAU physical and mental health outcomes, including mortality, over 2 years. POINT study results will likely be a key source in comparing and contrasting our results; however, our focus on depression distinguishes Pathways from the POINT study.

A unique component of the Pathways study is that it is beginning three years after the CDC opioid prescribing guidelines were published [63]. Since this landmark publication, the number of new opioid prescriptions dispensed annually has decreased, many health care systems and State governments have placed limitations on the duration of opioid prescriptions for new pain patients and the prevalence of tramadol dispensing has substantially increased. At first this would seem to reduce our ability to recruit informative subjects, however, we decided against trying to create a cohort that was characteristic of chronic opioid use 5 to 10 years ago. By allowing patients starting tramadol into the cohort and allowing multiple, short duration prescriptions to accumulate to 60 days of opioid exposure, our sample will enable us to collect data from patients exposed to current prescribing patterns. Results will be relevant to contemporary clinical care and with the large increase in tramadol use, we expect to report some of the first evidence for or against a relationship between this medication and depression and opioid misuse phenotypes.

The results from Pathways will impact several significant public health problems: chronic pain, the opioid epidemic, opioid misuse and use disorder and depression with and without comorbid psychiatric disorders. A crucial implication of our research to date is that NDE occurs in patients adherent to routine prescription OAU, free of opioid misuse and occurs in mid-life, beyond the usual age onset for depressive disorders. Our research to date suggests that these patients have no recent history of depression (i.e., no diagnoses for 2 years). However we believe the story is more complicated and this proposal will identify pre-OAU (e.g., prior depression/dythymia) and/or post-OAU (e.g., impaired sleep, poor physical and social functioning) risk factors for NDE. Results will provide an empirical basis for patient-provider risk-benefit discussions. If patients with no previous history of psychopathology are as vulnerable for OAU-related affective disorder as those about whom physicians normally have heightened awareness, (e.g., patients with opioid misuse or history of substance abuse), then clinicians must highlight this risk before prescribing. If non-OAU risk factors are found, then a more patient-centered approach that targets high risk patients is warranted. Starting an antidepressant may be warranted which may facilitate OAU tapering [64]. Our research will clarify when and how depression develops after OAU so that patients and providers can have informed discussions about reasonable opioid benefits and vulnerability to NDE.

## CONCLUSIONS

Using medical record data from 2000 to 2013, we have found duration of opioid use is associated with NDE, depression recurrence and worsening depression [2–4,15,16,18] Our cohort study will reveal whether this observation is accurate or better described as chronic OAU leading to anhedonia, vital exhaustion or dysthymia. We will inform safe opioid prescribing by elucidating those patients most at risk for adverse mental health outcomes and by identifying the pre-existing mental health phenotypes most strongly associated with developing opioid misuse, abuse and dependence. Last, our study will determine if the OAU-depression association is independent of pain related functional impairment, low social support and impaired sleep. If we find chronic OAU is significantly associated with NDE, even after controlling for the host of confounding factors shown in Table 1, then implementing training to screen for depression repeatedly during opioid pain management and treating depression to remission should become a central feature of safe opioid therapy.

## Figures and Tables

**Table 1.** Summary of survey instruments.

Constructs	Instrument/Measure	Recall period/When measured	Source
**Chronic Pain, severity, interference, duration, recency**	Brief Pain Inventory-10 item	Past 30 days/Baseline, 6-month, 12-month follow-up	[25]
**Pain severity/functioning**	PEGS	Past week/12 monthly surveys	[26]
**Painful conditions**	Electronic medical record (EMR) data: arthritis, musculoskeletal, back pain, neuropathy, headache	Medical record data from 1 year before baseline through 12-month follow-up	Not applicable
**Opioid Medication Use**	OAU medication names, dose, instructions for use, how really taken Self-report prescription opioid use before age 18	Current/Baseline, 6-month, 12-month follow-up Also collected in each of the 12 monthly surveys	Study experts in clinical pain management and opioid research developed assessment
**Non-opioid pain treatments**	Interest in psychological trx Physical therapy Chiropractic care cannabidiol	Lifetime/Baseline, 6-month, 12-month follow-up	Exploratory single item questions
**co-medications**	Medial record data on non-opioid analgesics, Benzodiazepines, corticosteroids, disease-modifying anti-rheumatic drugs, sedative hypnotics, other psychotropics criptions	Medical record data from 1 year before baseline through 12-month follow-up	Not applicable
**Opioid misuse**	Prescribed opioid difficulties scale (PODS) COMM Current Opioid Misuse Measure (COMM) Self-report of buprenorphine for OUD	Past 2 weeks and past year/Baseline, 6-month, 12-month follow-up Past 30 days/Baseline, 6-month, 12-month follow-up Lifetime–Baseline, 6-month, 12-month follow-up	[27,28] Single item exploratory
**Lifetime and past 12-month DSM-4 opioid use disorder**	SSAGA RedCap	lifetime and past 12 months, date onset and recency/Baseline and 12-month follow-up	[23]
**Lifetime and past year DSM-4 major depressive episode, dysthymia and mania/bipolar**	SSAGA RedCap	lifetime and past 12 months, date onset and recency/Baseline and 12-month follow-up	
**Depression symptoms**	PHQ-9 current	12 monthly surveys	[29]
**Anhedonia**	Snaith-Hamilton Pleasure Scale (SHAPS)	Past few days/Baseline, 6-month, 12-month follow-up	[30,31]
**Vital Exhaustion**	Maastricht Vital Exhaustion brief form	Current/ Baseline, 6-month and 12-month follow-up	[32]
**Depression treatment**	EMR antidepressants, referral to psychotherapy	Medical record data from 1 year before baseline through wave 3	Not applicable
**DSM-4 illicit drug use disorders**	SSAGA RedCap	lifetime and past 12 months, date onset and recency/Baseline and 12-month follow-up	[23]
**Alcohol misuse**	AUDIT-C	Lifetime and current/Baseline, 6-month, 12-month follow-up	[33]
**Smoking**	BRFFS	Lifetime and current/Baseline, 6-month and 12-month follow-up	[34]
**Family history of SUD and depression**	Self-report of mother and father any SUD and depression	Lifetime/Baseline	Based on SSAGA items
**Trauma and PTSD**	PC-PTSD-5	Lifetime and past 30 days at Baseline, past 30 days only at Baseline, 6-month and 12-month follow-up	[35]
**Anxiety**	GAD-7	Past 2 weeks/Baseline, 6-month, 12-month follow-up	[36]
**Sleep**	Pittsburgh Sleep Quality Index	Past month/Baseline, 6-month, 12-month follow-up	[37]
**Sleep Apnea**	STOP Questionnaire	Current/Baseline, 6-month, 12-month follow-up	[38]
**Social support**	PROMIS SF v2.0–Emotional Support 4a–4 item social support	Current/Baseline, 6-month, 12-month follow-up	[39]
**Chronic health conditions related to depression**	EMR, ICD-9 and ICD-10 diagnoses for hypertension, heart disease, diabetes, hyperlipidemia, obesity	Medical record data from 1 year before baseline through 12-month follow-up	Not applicable
**social functioning**	PROMIS Ability to participate in social roles and activities	Current/Baseline, 6-month, 12-month follow-up	[40]
**Discounting**	3 item discounting instrument	Current/Baseline, 6-month, 12-month follow-up	[41]

**Sociodemographics–age, gender,** education, employment, income, marital status		Current/Baseline, 6-month, 12-month follow-up	not applicable
